# Size- and Time-Dependent Effects of Polyethylene Microplastics on Soil Nematode Communities: A 360-Day Field Experiment

**DOI:** 10.3390/toxics14020127

**Published:** 2026-01-29

**Authors:** Tianyao He, Shiyu Zhu, Xiankun Liu, Jie Chen, Liping He, Kehong Wang, Yihua Zhu, Hongzhi Xu

**Affiliations:** 1 College of Environmental and Chemical Engineering, Chongqing Three Gorges University, Chongqing 404100, China; ty_889512@163.com (T.H.); badada06030@163.com (S.Z.); lxk136631@163.com (X.L.); hlp_weird@163.com (L.H.); zyh001016@126.com (Y.Z.); xhz1884712@163.com (H.X.); 2 College of Ecology and Environment, Xinjiang University, Urumqi 833300, China; cj_7404@163.com; 3 Chongqing Observation and Research Station of Environment and Ecology in the Three Gorges Reservoir Area, Chongqing Three Gorges University, Chongqing 404100, China

**Keywords:** microplastic particle size, soil nematode diversity, polyethylene, complexity and stability, temporal dynamics

## Abstract

Soil ecosystems are seriously contaminated by microplastics of varying particle sizes, yet the ecological consequences across a broader size spectrum remain poorly understood. We conducted a 360-day field experiment to examine the effects of seven microplastic size fractions (ranging from 6.5 μm to 1000 μm) on the composition, trophic structure, temporal dynamics, complexity, and stability of soil nematode communities. Results showed that microplastics altered nematode community composition and structure, with impacts clearly dependent on both particle size and exposure time. Microplastics generally reduced the abundance, complexity, and stability of nematode communities, except for the 25 μm and 500 μm particles. Temporal analysis revealed an initial increase in nematode abundance, followed by a long-term decline across most treatments. Structural equation modeling indicated that microplastics regulated nematode diversity and stability through pathways that varied with particle size. We recommend that the environmental risk assessments for soil microplastics incorporate testing across a broad size spectrum and over extended timescales to capture their complex and dynamic impacts.

## 1. Introduction

Microplastic pollution has emerged as a global environmental crisis, with accumulating evidence demonstrating its pervasive distribution in soil ecosystems [1,2,3]. Recent studies have documented its adverse effects on soil fauna biodiversity, which are closely linked to their physical and chemical properties, such as concentration, shape, and polymer composition [4,5,6].

Particle size is a critical factor determining the ecological impacts of microplastics on soil organisms [7]. In soil, microplastics exist as a continuous mixture of various sizes resulting from physical fragmentation, chemical aging, and biodegradation [8,9]. Furthermore, their migration, adsorption, and retention dynamics in soils vary with particle size [10]. Therefore, assessing the impacts of microplastics on soil biodiversity requires understanding the differential effects across the particle size spectrum.

Soil fauna, including earthworms, springtails, and nematodes, exhibit distinct size-dependent behaviors in ingesting, retaining, accumulating, biodegrading, and excreting microplastics [11,12,13]. Generally, smaller particles exhibit higher environmental mobility, bioavailability, potential ecological toxicity, and trophic transfer rates, whereas larger particles have greater adsorption capacity and longer retention times [14,15,16]. However, current knowledge of size-dependent effects remains fragmented, as most studies overlook microplastic degradation processes and typically examine only two to three discrete particle sizes per experiment [11,17].

Nematodes are valuable bioindicators for soil ecosystem health as they play pivotal roles in organic matter decomposition, nutrient cycling, and microbial community regulation [18]. Substantial evidence has confirmed that microplastics detrimentally affect nematodes by inhibiting growth, reducing population densities, and altering trophic structure, with these impacts strongly mediated by particle size through distinct pathways [4,17]. Most studies suggested that smaller particles impaired nematode reproduction due to the size constraints of the buccal cavity [19]. However, some reports have presented contrasting findings. For example, Lin et al. [20] observed that 37 μm particles had no significant effects on bacterivorous and fungivorous nematode populations because they did not disrupt microbial food resources. Such inconsistencies, combined with the limited particle size ranges examined across studies, have prevented a scientific consensus regarding the overall impacts of microplastics on nematode communities.

Notably, research has been largely confined to controlled laboratory conditions with short observation [13,21], resulting in an incomplete assessment of long-term ecological impacts due to the failure to simulate complex natural ecosystem interactions. Moreover, field soils typically contain microplastics with continuous size distributions, resulting from ongoing photodegradation and biodegradation [22]. Therefore, a systematic evaluation of particle-size-dependent effects on nematode communities is imperative for comprehensive environmental risk assessment.

We conducted a 360-day field experiment with seven microplastic size treatments (6.5 μm, 25 μm, 75 μm, 150 μm, 250 μm, 500 μm, and 1000 μm) to analyze their impacts on soil nematode communities. The selection of these sizes was based on a field investigation by Xie et al. [23], which identified this range as the dominant spectrum for soil microplastics in the study region, accounting for over 75% of detected particle abundance. We aimed to address the following questions: (1) Do different microplastic sizes exert consistent impacts on the composition and structure of soil nematode communities? (2) How do the temporal dynamics and stability of soil nematode communities respond to microplastic size gradients? (3) What ecological mechanisms underlie the effects of different microplastic sizes on soil nematode communities? By integrating size-specific community analyses, our findings provided mechanistic insights into microplastic-driven disruptions of the soil ecosystem.

## 2. Materials and Methods

### 2.1. Study Area

The field experiment was conducted at the Chongqing Observation and Research Station of Environment and Ecology in the Three Gorges Reservoir Area (108.50° E, 30.89° N). This region has a subtropical monsoon climate, with an average annual temperature of approximately 16.8 °C and annual precipitation of around 1125 mm [24]. The study site predominantly featured *Citrus reticulata* plantations interspersed with patchy farmlands cultivating *Zea mays*, *Ipomoea batatas*, and various *vegetable species*. To minimize potential confounding factors, we specifically selected abandoned farmland plots that had been free from agricultural plastic film and chemical applications, thereby ensuring baseline microplastic contamination levels. Based on the investigation by Xie et al. [23], the concentration of soil microplastics was 960 particles/kg, dominated by polyamide (66%). The morphologies were primarily fibers and fragments, with size distributions as follows: >2000 μm (7%), 1000–2000 μm (16%), 500–1000 μm (22%), 200–500 μm (25%), 100–200 μm (14%), and <100 μm (16%). Detailed soil physicochemical properties are presented in Table 1.

### 2.2. Processing and Sampling

Before the experiment began, surface soils (0 to 20 cm) were collected, homogenized by sieving through a 1 cm mesh, and thoroughly mixed to ensure uniformity. Granular polyethylene microplastics were sieved through 16, 32, 60, 100, 200, 500, and 2000 meshes, yielding seven size fractions: 6.5 μm, 25 μm, 75 μm, 150 μm, 250 μm, 500 μm, and 1000 μm. Microplastics of each size were washed with distilled water and dried at 40 °C.

The target microplastic concentration was deliberately set at approximately 0.01% (*w*/*w*), reflecting realistic pollution levels reported in agricultural soils (e.g., Xie et al. [23]) and supported by ecotoxicological evidence showing that such concentrations of polyethylene can affect soil nematodes [4]. To achieve this, a total of 0.3 m^3^ of prepared soil was mixed with 40 g of microplastics using the following protocol: 40 g of microplastics were first blended with 2 L of soil to obtain a homogeneous pre-mix, which was then progressively diluted with 10 L and 50 L of soil, and subsequently amalgamated with the remaining soil. The entire mixture was passed three times through a 1 cm sieve to ensure uniform distribution. The final mixture was mounded into a 1 m × 1 m plot (Figure S1). Plots were spaced 0.5 m apart, with five replicates per microplastic treatment. In addition, five control plots (CK) without microplastics were established. All seven microplastic treatments and the control were arranged using a randomized block design.

Soil samples were collected at 60, 120, 180, and 360 days after microplastic addition to capture short-term response, mid-term trends, and long-term effects. Six soil cores (0 to 10 cm) were sampled per plot using a 3 cm diameter auger. Cores from the same plot were composited and transported to the laboratory. Each sample was divided into two subsamples: one for soil nematode extraction and the other for soil property analysis after air-drying.

### 2.3. Soil Nematode Extraction and Identification

Nematodes were extracted from 100 g of fresh soil using the sucrose centrifugation method [25]. The number of individuals was standardized based on measured soil moisture. Extracted nematodes were identified to the genus level according to Zhang et al. [26]. All nematodes were classified into herbivores, bacterivores, fungivores, and omnivore-predators [27]. The community structure of soil nematodes was quantified by abundance, genus richness, Shannon−Wiener index (H’), and Pielou’s evenness index (J’).

### 2.4. Soil Physicochemical Analysis

Soil moisture (SM) was determined by drying fresh soil at 105 °C to a constant weight. Soil pH was measured in a 1:2.5 (*w*/*v*) soil-deionized water suspension. Soil organic carbon (SOC) was quantified using the K_2_Cr_2_O_7_ oxidation-spectrophotometry method. Total nitrogen (TN) was determined by the alkaline K_2_S_2_O_8_ spectrophotometric method. Dissolved organic carbon (DOC) and nitrogen (DON) were extracted with a 0.5 M K_2_SO_4_ solution and tested using a TOC/N analyzer (Shimadzu TOC-V, Kyoto, Japan). Microbial biomass carbon (MBC) and nitrogen (MBN) were determined by the chloroform fumigation−potassium sulfate extraction method. The activities of sucrase (SUC), urease (URE), and alkaline phosphatase (ALP) were measured colorimetrically using dinitrosalicylic acid, sodium phenolate−sodium hypochlorite, and sodium benzoyl phosphate, respectively [28,29,30]. Plant biomass (PB) was assessed using the harvest method.

### 2.5. Statistical Analyses

**Figure 1:**
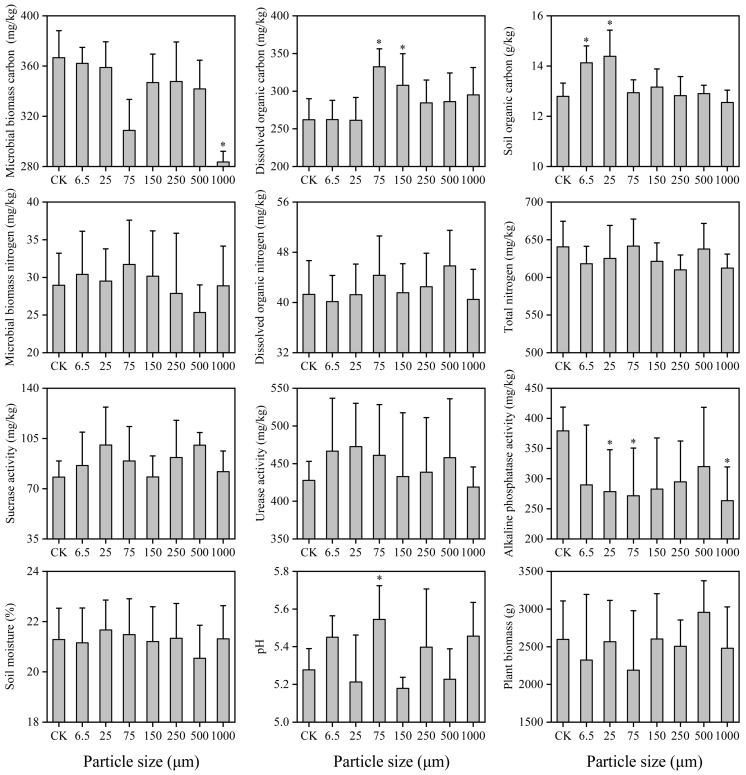
Effects of microplastic particle size on environmental factors. * denotes significant differences between microplastic treatments and the control.

**Figure 2:**
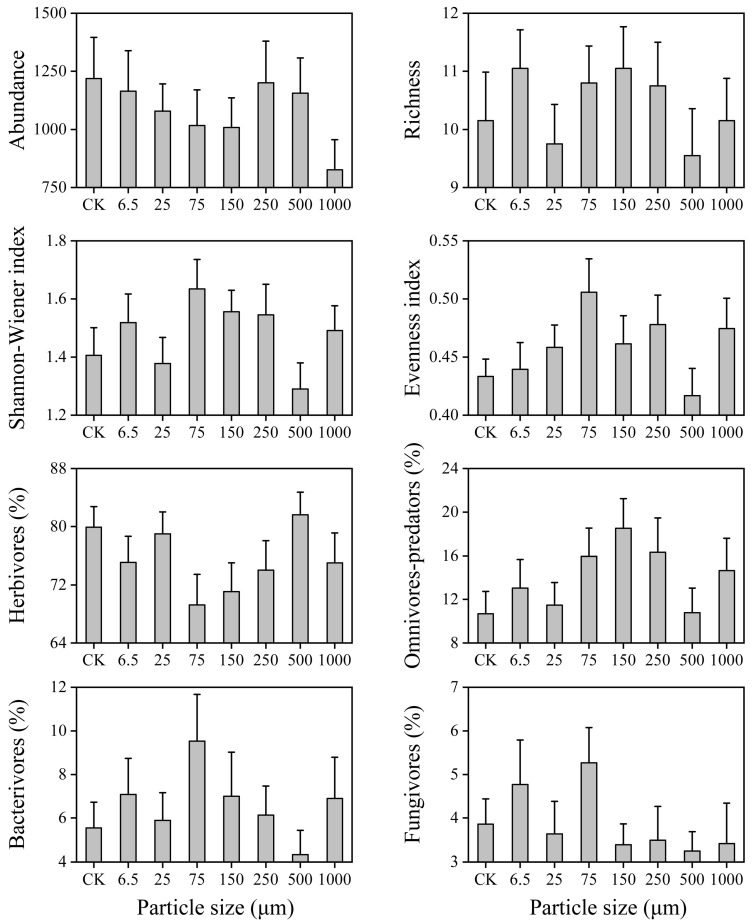
Abundance, richness, Shannon−Wiener index, evenness, and the relative abundance of the four trophic groups of soil nematodes under different microplastic size treatments.

A two-way ANOVA was used to test the effects of microplastic particle size and sampling time on nematode abundance, richness, Shannon−Wiener index (H’), Pielou’s evenness index (J’), the relative abundance of each trophic group, and soil physicochemical properties. Prior to ANOVA, the homogeneity of variances was verified using Mauchly’s test. When the assumption was violated, data were log(x + 1) or square-root-transformed. Post hoc comparisons were conducted using Fisher’s Least Significant Difference (LSD) test. Microplastic size effects and temporal dynamics were tracked by comparing each treatment to the concurrent control, which served as the experimental baseline. When analyzing the effects of different microplastic particle sizes on environmental factors and soil nematode community structure metrics (Figure 1 and Figure 2), five replicates were averaged across sampling times.

Permutational multivariate analysis of variance (PERMANOVA) was used to determine the effects of microplastic particle size and sampling time on soil nematode community composition [31]. Post hoc comparisons were conducted using a Monte Carlo permutation test with Bonferroni correction [32]. Because the DCA axis length was <3.0, redundancy analysis (RDA) was used to examine relationships between nematode community composition and environmental factors [32]. The significance of each environmental factor was also tested using Monte Carlo permutation tests.

Co-occurrence network analysis of soil nematode communities was conducted to evaluate structural complexity and stability [33]. Before analysis, genera with a relative abundance < 0.01% were removed. Spearman correlations were calculated based on random matrix theory, and statistical significance was determined using the Benjamini–Hochberg correction [34]. Quantified network properties included the number of nodes and edges, average path length and degree, and network diameter and modularity [35]. Stability was calculated based on the network cohesion, determined by the closeness between nodes and the absolute value of the ratio of positive to negative cohesion [36,37].

Structural equation modeling (SEM) was conducted to examine the causal relationships between microplastics and soil nematode communities. A piecewise SEM approach was implemented to accommodate the hierarchical data structure, with model fit assessed using Shipley’s test of directional separation [38]. To optimize model parsimony, non-significant pathways (*p* > 0.05) were iteratively removed through stepwise model reduction. All above analyses were performed in R software (4.3.0 version) using the ‘igraph’, ‘Hmisc’, ‘vegan’, and ‘piecewiseSEM’ packages.

## 3. Results

### 3.1. Soil Properties

Significant alterations were observed in MBC, SOC, DOC, and pH (*p* < 0.05), whereas SM, PB, MBN, DON, SUC, URE, and ALP showed no statistically significant responses (*p* > 0.05). The results demonstrated size-dependent effects of microplastics on soil properties (Figure 1). All microplastic treatments reduced MBC, TN, and ALP, while SUC and DOC increased. SOC showed an increasing trend except in the 1000 μm treatment. MBN increased in treatments with microplastic sizes ≤ 150 μm but decreased in others. DON increased in the 75–500 μm treatments and decreased in others. All microplastic treatments increased URE, except for the 1000 μm treatment. Microplastics increased SM (except for in the 6.5 μm, 150 μm, and 500 μm treatments) and pH (except for in the 25 μm,150 μm, and 500 μm treatments). PB was reduced by microplastics, except for in the 150 μm and 500 μm treatments.

### 3.2. Soil Nematode Community Composition

A total of 173,416 individuals belonging to 58 genera were recorded (Table S1). The dominant taxa were *Helicotylenchus* and *Paratylenchus*, representing 38.9% and 35.3% of all individuals, respectively. Common taxa, including *Filenchus* and 11 other genera, collectively accounted for 18.2%. Rare taxa comprised 45 genera, representing 7.6%. Herbivores, omnivore-predators, bacterivores, and fungivores comprised 16, 11, 21, and 10 genera, accounting for 75.4%, 14.0%, 6.6%, and 4.0% of all individuals, respectively. PERMANOVA showed that microplastics had significant effects on nematode community composition (*p* = 0.011). Pairwise comparisons revealed significant differences between all treatment groups, except between the 500 μm and 1000 μm treatments.

### 3.3. Soil Nematode Community Structure

Microplastics significantly affected the Shannon−Wiener index, but no significant effects were observed on nematode abundance, genus richness, or evenness index (Table 2). Nematode abundance decreased across all microplastic treatments (Figure 2), suggesting a general negative effect. Treatments with 6.5 μm, 75 μm, 150 μm, 250 μm, and 1000 μm microplastics increased nematode richness and the Shannon−Wiener index, whereas the 25 μm and 500 μm treatments decreased them (Figure 2). All microplastic treatments increased the evenness index, except for the 500 μm treatment (Figure 2).

Microplastics significantly affected the relative abundance of herbivores and omnivore-predators, but not bacterivores and fungivores (Table 2). All microplastic treatments decreased the relative abundance of herbivores and increased that of bacterivores, except for 500 µm (Figure 2). The relative abundance of omnivore-predators increased in all treatments (Figure 2). The 6.5 µm and 75 µm microplastics increased the relative abundance of fungivores, while other sizes decreased it (Figure 2). These results revealed size-dependent effects of microplastics on nematode community trophic structure.

### 3.4. Temporal Dynamics

**Figure 3:**
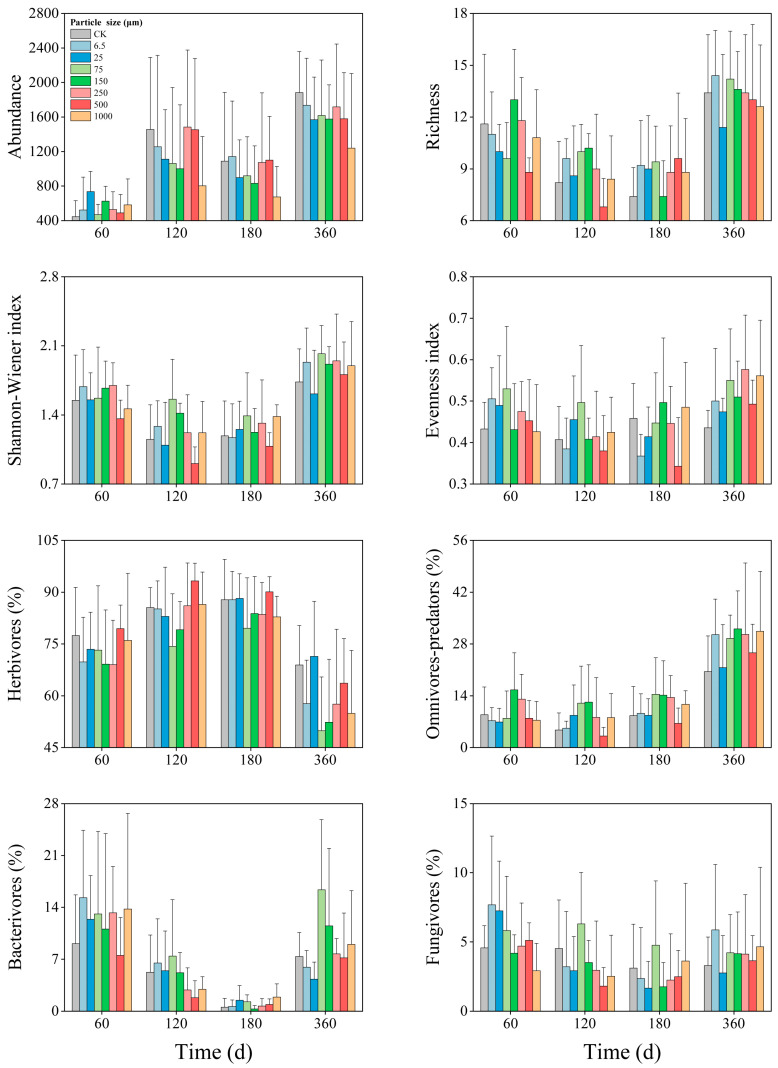
Temporal dynamics of soil nematode community indices (abundance, richness, Shannon−Wiener index, and Evenness index) and the relative abundance of four trophic groups under different microplastic treatments.

The results demonstrated the time-varying effects of microplastics on soil nematode communities (Figure 3, Table 2). In general, all microplastic treatments increased nematode abundance at 60 days but decreased it from 120 to 360 days. With the exception of the 150 μm and 250 μm treatments, microplastics generally decreased nematode richness at 60 days. At 120 and 180 days, most treatments (excluding 500 µm) increased nematode richness. At 360 days, 25 µm, 500 µm, and 1000 µm microplastics decreased nematode richness, while others increased it. Both the Shannon−Wiener and evenness indices of nematode communities fluctuated and generally increased across the observation period in all microplastic treatments except 25 μm and 500 μm. Over time, the relative abundance of herbivores decreased, while that of omnivore-predators increased (Figure 3). The relative abundance of bacterivores and fungivores increased at 60 days, decreased between 120 and 180 days, and rose again at 360 days (Figure 3).

### 3.5. Relationships Between Environmental Factors and Soil Nematodes

**Figure 4:**
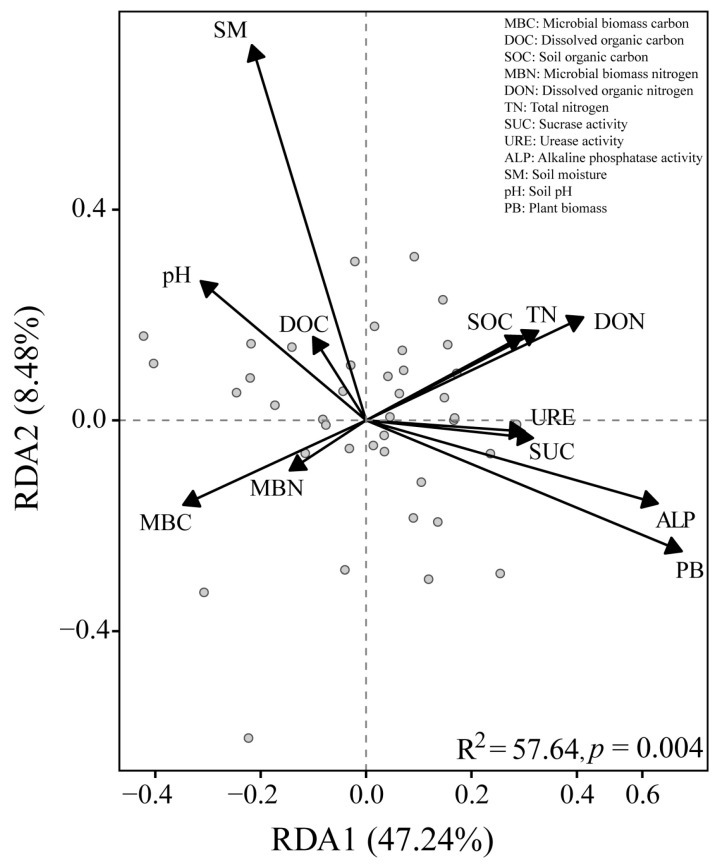
Redundancy analysis of soil nematode communities and environmental factors.

The results of the RDA revealed that measured environmental factors collectively explained 57.64% of the variation in nematode community composition (Figure 4). Among these, MBC and PB had significant effects on soil nematode communities (*p* < 0.01), contributing 20.64% and 14.46% of the total variation, respectively (Table S4). The first two RDA axes together explained 55.72% of the total variation (axis 1: 47.24%, axis 2: 8.48%; *p* < 0.01). This indicated that MBC and PB are key factors impacting soil nematode community composition.

### 3.6. Nematodes Co-Occurrence Network Analysis

**Figure 5:**
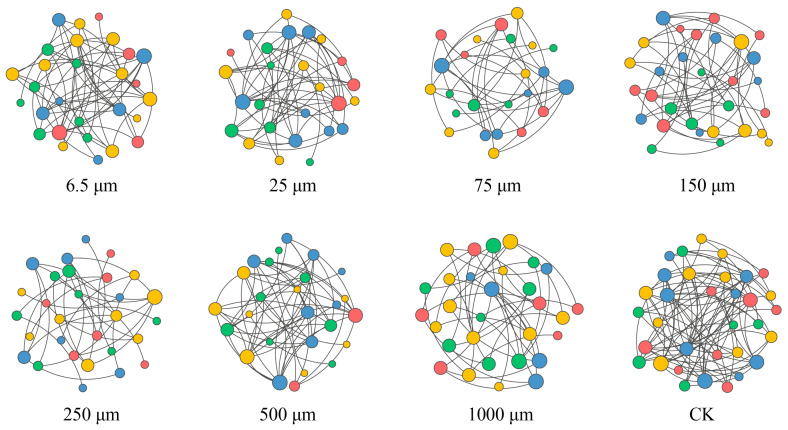
Co-occurrence networks of soil nematode communities under different microplastic treatments. Red, yellow, green, and blue represent herbivores, bacterivores, fungivores, and omnivore-predators, respectively.

Network analysis revealed significant structural modifications in nematode communities induced by microplastics (Figure 5). All microplastic treatments reduced network complexity compared to the control, as reflected by decreases in the number of nodes and edges, average degree, network density, and modularity, and increases in network diameter and average path length (Table 3). Compared to the control, microplastics of 75 μm, 250 μm, and 1000 μm induced more pronounced community disruption. Moreover, the topology of nematode networks under microplastic treatments varied with sampling time (Figure S2). At 60 days, microplastics of 6.5 μm, 500 μm, and 1000 μm exhibited the strongest negative effects. Notable impacts were observed for 150 μm and 500 μm particles at 180 days, while 25 μm, 75 μm, and 250 μm particles exerted the dominant influence at 360 days. Interestingly, except for 6.5 μm and 500 μm, microplastics enhanced community complexity at specific time points. In terms of community stability, only 25 μm and 500 μm particles had a stabilizing effect, while all others had a destabilizing effect (Table 3).

### 3.7. Effects of Microplastics on Nematode Diversity and Stability

**Figure 6:**
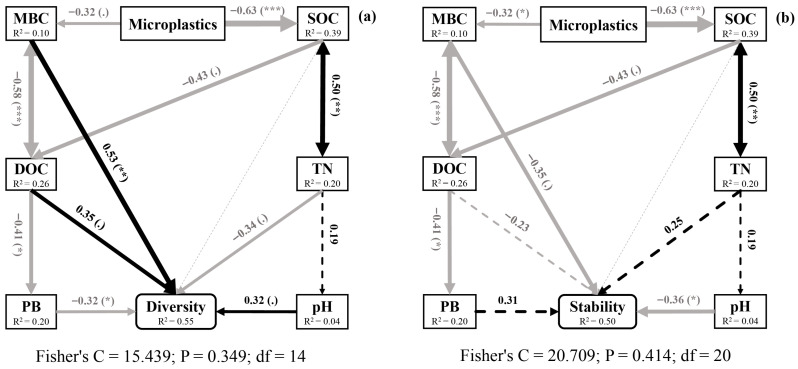
Structural equation model describing the effects of microplastics on community diversity (**a**) and stability (**b**) of soil nematode communities. (.): *p* < 0.1, *: *p* < 0.05, **: *p* < 0.01, and ***: *p* < 0.001.

Structural equation modeling (SEM) revealed complex mechanistic pathways through which microplastics influenced nematode communities (Figure 6). Despite PB and TN decreasing community diversity, microplastics indirectly enhanced soil nematode diversity by decreasing MBC and DOC while increasing pH (Figure 6a). However, microplastics also indirectly reduced soil nematode stability by decreasing MBC and DOC and increasing pH (Figure 6b). Although the effect transmitted from SOC through TN and PB to stability was marginally positive, this compensatory mechanism failed to offset the net destabilizing impact. These findings collectively indicated that microplastics altered nematode communities through integrated biotic−abiotic interactions. SEM analysis further revealed distinct pathways through which different microplastic sizes influenced the diversity and stability of soil nematode communities compared to the control (Figure S3).

## 4. Discussion

### 4.1. Microplastics Reshaped Soil Nematode Community Structure

Our study found that microplastics significantly altered the composition of soil nematode communities, which is consistent with most recent studies [20,39]. The observed shifts likely resulted from a combination of direct and indirect pathways. Nematodes can selectively ingest microplastics depending on their feeding strategies, stylet morphology, and mobility [5,21]. Although the microplastic sizes used in this study generally exceeded the direct ingestion capacity of most nematode species [40], we still observed a decrease in soil nematode abundance (Figure 2). This suggested that microplastics may have fragmented into smaller particles, which could induce oxidative damage and intestinal blockage [21]. Additionally, the release of polymer monomers from aged microplastics may contribute to nematode toxicity [4], corroborated by declines in plant and microbial biomass (Figure 1). Furthermore, microplastics could indirectly affect soil nematodes by altering physicochemical properties, enzyme activities, and microbial-derived food resources (Figure 1), all of which are key factors shaping soil nematode communities [41]. The observed decreases in plant and microbial biomass indicated a reduction in the habitat quality and food resource availability for nematodes [42].

Most microplastic treatments decreased nodes, edges, average degree, network density, and modularity, indicating a loss of species and interspecific interactions (Table 3; Figure 5). Concurrently, increases in network diameter and average path length suggested that species interactions became less efficient, potentially slowing energy flow within the community [35]. In addition, decreases in the diversity, complexity, and stability of soil nematode communities aligned with the established literature [43]. Recent studies have reported that microplastics reduce nematode abundance and diversity by inhibiting movement, foraging, and reproduction [44,45]. For example, Yang et al. [39] documented that 200 μm polypropylene microplastics significantly lowered nematode abundance and richness in a maize soil after three months. Similarly, Liu et al. [43] found that polyethylene microplastics negatively affected the community complexity and stability of soil nematodes in a 2-month experiment, pointing out that microplastics affect soil nematode communities by modifying microbial abundance and activity, plant root distribution and productivity, and soil physicochemical characteristics [45,46]. Community restructuring was particularly evident in herbivorous nematodes, accompanied by compensatory increases in omnivore-predators and bacterivores (Figure 2). Although herbivores are unlikely to directly ingest large microplastics due to their specialized stylets [40], microplastics may affect them indirectly by altering their basal food resources [47], such as PB and MBC (Figure 1).

### 4.2. The Effect of Microplastics on Soil Nematodes Depended on Particle Size

Our results demonstrated a nonlinear size-dependent response of nematode community structure and dynamics to microplastics (Figure 2). In contrast to some studies reporting stronger effects from smaller microplastics [44], we found that 6.5 μm, 75 μm, 150 μm, and 250 μm microplastics generally increased Shannon diversity, whereas 25 μm and 500 μm exhibited the opposite trend (Figure 3; Table 3). In addition, only the 500 µm treatment reduced the evenness index of soil nematodes and the relative abundance of bacterivorous nematodes, while increasing the relative abundance of herbivorous nematodes compared to the control treatment (Figure 2). This nonlinear pattern may result from trade-offs between interspecific and intraspecific interactions [48], leading to differential cascading effects on bacterivores and fungivores not captured by our metrics. Microplastics significantly modified interactions between nematodes and other soil organisms that served as trophic resources, competitors, predators, and habitat providers [6]. For example, 6.5 μm and 75 μm microplastics enhanced fungivore abundance (Figure 2), suggesting microplastic size modulates interactions between microorganisms and nematodes. However, the field experiment included uncertain variations in soil mixing uniformity, aggregation structure, initial nematode communities, and microhabitat conditions, which may have resulted in some discrepancies, such as differences in the effects of 25 μm and 500 μm microplastics [9]. While these factors contribute to inconsistent trends, we emphasized the substantial challenges that microplastic particle size poses to environmental risk assessment.

Another possible explanation involved the particle-size-dependent modification of soil properties, which differentially constrained various nematode species or trophic groups, thereby cascading to affect nematode diversity, complexity, and stability [39]. Specifically, 75 μm microplastics preferentially favored bacterivorous and fungivorous nematodes by significantly increasing DOC and pH, along with stimulating microbial activity. The 150 μm microplastics improved soil aggregation and SM, which temporarily benefited omnivore-predator nematodes while ultimately decreasing food web complexity [49]. Conversely, 1000 μm microplastics reduced soil pore connectivity and MBC, disproportionately impacting herbivores and ultimately destabilizing communities. Such nonlinear responses suggested the necessity to characterize both the distribution patterns and temporal dynamics of microplastic particle sizes in soil for accurate risk assessments.

### 4.3. Pathways of Microplastics Affected Soil Nematode Communities

Microplastics influenced soil nematode communities via multiple pathways, primarily by altering soil properties and nutrient availability (Figure 4). They reduced microbial biomass carbon (MBC) and plant biomass (PB), thereby disrupting nematode food resources and habitat conditions [50,51]. Microplastic-induced shifts in microbial composition stimulated bacterivorous and fungivorous nematodes, which subsequently regulated higher trophic levels through micro-food web interactions [46,48]. The negative effect on PB further triggered bottom-up trophic cascades, directly impacting herbivores and indirectly influencing free-living nematodes [52].

These impact pathways varied significantly with microplastic size and sampling time (Figure S3). The 6.5 μm particles inhibited microbial activity, raised soil pH and SOC, and ultimately reduced community stability. In contrast, 25 μm and 500 μm microplastics enhanced food resource availability by stimulating sucrase activity, thereby improving stability. The 25 μm particles likely enhanced stability by stimulating sucrase-producing microbes through direct physicochemical interactions, facilitated by their relatively high mobility and specific surface area in the soil matrix [15]. In comparison, the 500 μm microplastics acted as persistent physical substrates, gradually releasing bioavailable carbon via surface aging and thereby supporting a more stable and prolonged supply of resources to the soil food web [44,53]. The divergent pathways highlighted that the effects of microplastics on soil fauna were governed by a complex trade-off between environmental factors and organism behavior [11]. Furthermore, microplastics induced dynamic community responses. Nematode abundance and richness initially increased but declined with prolonged exposure. Although transient positive effects were observed, long-term impacts were predominantly negative. This temporal shift can be attributed to the gradual depletion of key soil factors, such as MBC, coupled with the progressive fragmentation of microplastics into more bioavailable and toxic sizes [53,54]. Over time, these processes disrupt energy flow through the soil food web, ultimately overriding any short-term benefits and leading to a decline in nematode abundance and community stability [45].

### 4.4. Effect of Microplastics on Soil Nematodes Depended on Exposure Time

Our study emphasized that microplastics induced temporally divergent effects on soil nematode communities. For example, nematode abundance increased at 60 days but declined after 120 days, while richness rose at 120 and 180 days but decreased at 60 and 400 days (Figure 3). Although microplastics had temporarily positive effects on nematodes during specific periods, our results strongly underscored the long-term negative effects. Critically, microplastics can break into smaller and variably shaped particles through physical, chemical, and biological processes [9]. The ecological impacts of microplastics on nematodes evolve with aging time, particle size distribution, and shape heterogeneity, which determine their environmental fates, degradation kinetics, and biological interactions [21,53].

At 60 days, the positive effects were observed in 6.5 μm, 500 μm, and 1000 μm microplastic treatments, suggesting both ingestible smaller particles and larger particles that cause physical disturbance jointly drive the early ecological stress [14,40]. Mueller et al. [13] have reported that short-term exposure to microplastics accelerated the growth of certain nematode populations. By 180 days, the dominant effects shifted to the 150 μm and 500 μm particles, reflecting the aging processes of microplastics in the soil environment. Existing studies have demonstrated that toxicity to nematodes persisted with aged polyethylene microplastics [53]. However, at 360 days, the strongest negative effects were attributed to the smaller particles, specifically 25 μm, 75 μm, and 250 μm (Figure S2). This suggested that in the long term, smaller particles may present greater risks because of their higher potential for ingestion, bioaccumulation, and interaction with biota [55,56]. This pattern indicated that the ecological impacts of microplastics changed over time, likely influenced by various size-dependent mechanisms, including initial physical disruption and fluctuating bioavailability [57,58]. These findings highlighted that research on the ecological effects of microplastics should rely on long-term observational studies rather than acute toxicity testing.

## 5. Conclusions

In summary, our 360-day field experiment demonstrated that the ecological impacts of microplastics on soil nematodes were strongly dependent on both particle size and exposure duration. Despite transient positive effects for certain size classes, the overall impact on nematode communities was negative. However, the pathways by which different particle sizes of microplastics affect soil nematodes are inconsistent. Microplastics primarily exerted negative impacts by reducing soil microbial biomass carbon and plant biomass. In addition, the ecological impacts of microplastics on nematodes evolved with exposure time. Consequently, risk assessments based on single-size fractions or short-term laboratory studies may underestimate or misrepresent the complexity of these impacts in natural settings. Our results strongly suggest that current risk assessment frameworks are inadequate for evaluating microplastic pollution in soils. We argue that future standards should incorporate particle size distribution, aging processes, and the legacy effects of microplastics in soils as mandatory parameters to accurately assess the environmental risks posed by these contaminants.

## Figures and Tables

**Table 1:** Soil physicochemical properties of the study site.

Soil Properties	pH	Soil Moisture (%)	Soil Organic Carbon (g/kg)	Total Nitrogen (mg/kg)	Dissolved Organic Carbon (mg/kg)	Dissolved Organic Nitrogen (mg/kg)	Microbial Biomass Carbon (mg/kg)	Microbial Biomass Nitrogen (mg/kg)
Content	5.13	13.00	12.8	654.3	122.8	38.8	307.2	17.6

**Table 2:** Results of a two-way ANOVA testing the effects of microplastic particle size and sampling time on nematode abundance, richness, the Shannon−Wiener index (H’), the evenness index (J’), and the relative abundance of each trophic group.

Measured Metrics	Microplastic		Sampling Date		Microplastic × Sampling Date	
	F	*p*	F	*p*	F	*p*
Abundance	0.97	0.453	22.52	0.000	0.30	0.999
Richness	0.91	0.499	24.17	0.000	0.73	0.796
Shannon−Wiener index	2.22	0.036	31.41	0.000	0.47	0.976
Evenness index	1.57	0.149	6.67	0.000	0.81	0.701
Herbivores (%)	2.23	0.036	34.52	0.000	0.45	0.982
Omnivores-predators (%)	2.33	0.029	47.73	0.000	0.40	0.991
Bacterivores (%)	1.24	0.287	24.62	0.000	0.71	0.819
Fungivores (%)	0.98	0.448	4.20	0.007	0.65	0.876

**Table 3:** Topological characteristics of the co-occurrence networks of soil nematode communities under different treatments.

Network Metrics	CK	6.5 μm	25 μm	75 μm	150 μm	250 μm	500 μm	1000 μm
Number of nodes	33	28	26	24	30	29	26	30
Number of edges	78	50	49	32	44	30	54	40
Average degree	4.73	3.58	3.77	2.67	2.93	2.07	4.15	2.67
Network diameter	2.81	3.72	5.04	2.98	4.60	4.46	2.98	3.27
Average path length	1.37	1.56	1.70	1.34	1.93	1.96	1.36	1.56
Network density	0.15	0.13	0.15	0.12	0.10	0.07	0.17	0.09
Modularity	0.84	0.82	0.74	0.81	0.81	0.77	0.77	0.71
Stability	6.92	5.35	7.94	5.15	5.14	6.05	8.68	5.41

## Data Availability

The original contributions presented in this study are included in the article/Supplementary Materials. Further inquiries can be directed to the corresponding author.

## Supplementary Materials

The following supporting information can be downloaded at https://www.mdpi.com/article/10.3390/toxics14020127/s1: Figure S1. Schematic diagram showing the layout of the experimental farmland plots (1 m × 1 m); Figure S2: Co-occurrence network analysis of soil nematode communities across different microplastic treatments and sampling times. Red, yellow, blue, and green represent herbivores, bacterivores, fungivores, and omnivore-predators, respectively; Figure S3: Structural equation modeling revealed divergent pathways through which microplastics of different particle sizes regulate nematode community diversity and stability. (.): *p* < 0.1, *: *p* < 0.05, and **: *p* < 0.01. Table S1: Taxonomic composition of soil nematode communities at the genus level under different microplastic treatments. Table S2. Means ± SD of soil nematode community metrics under different microplastic particle sizes and sampling times. Table S3. Mean ± SD of environmental factors under different microplastic particle size treatments. Table S4 Explanatory of environmental factors for the variation in soil nematode community composition.
