# Supplementary material for: Size- and Time-Dependent Effects of Polyethylene Microplastics on Soil Nematode Communities: A 360-Day Field Experiment
Source: Toxics. 2026 Jan 29;14(2):127. doi: 10.3390/toxics14020127 (PMC12944334; doi:10.3390/toxics14020127)
Supplement: Supplementary file 1 [file toxics-14-00127-s001.zip › toxics-4106333-supplementary.pdf]

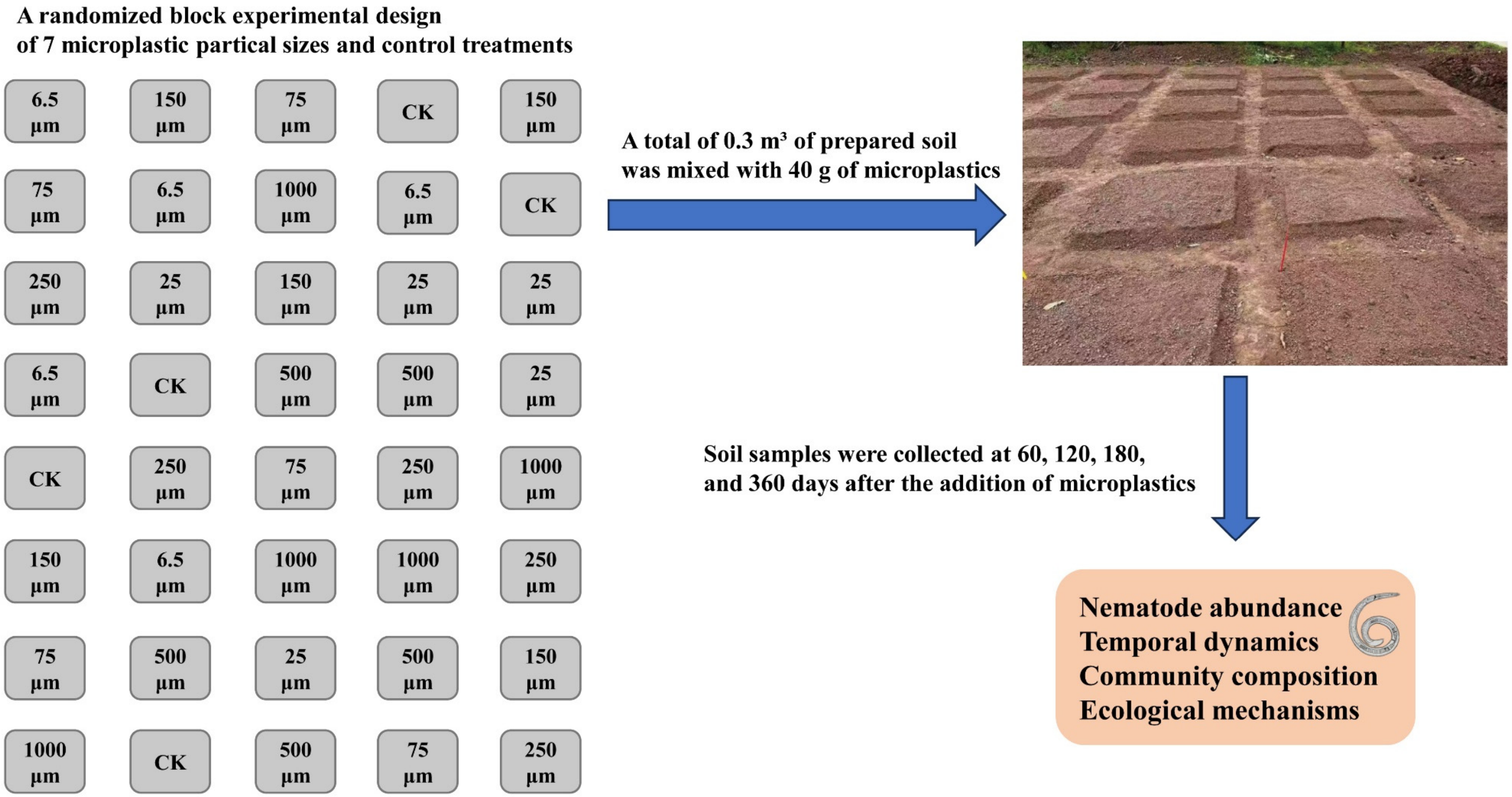


**Figure S1.** Schematic diagram showing the experimental design and plots.

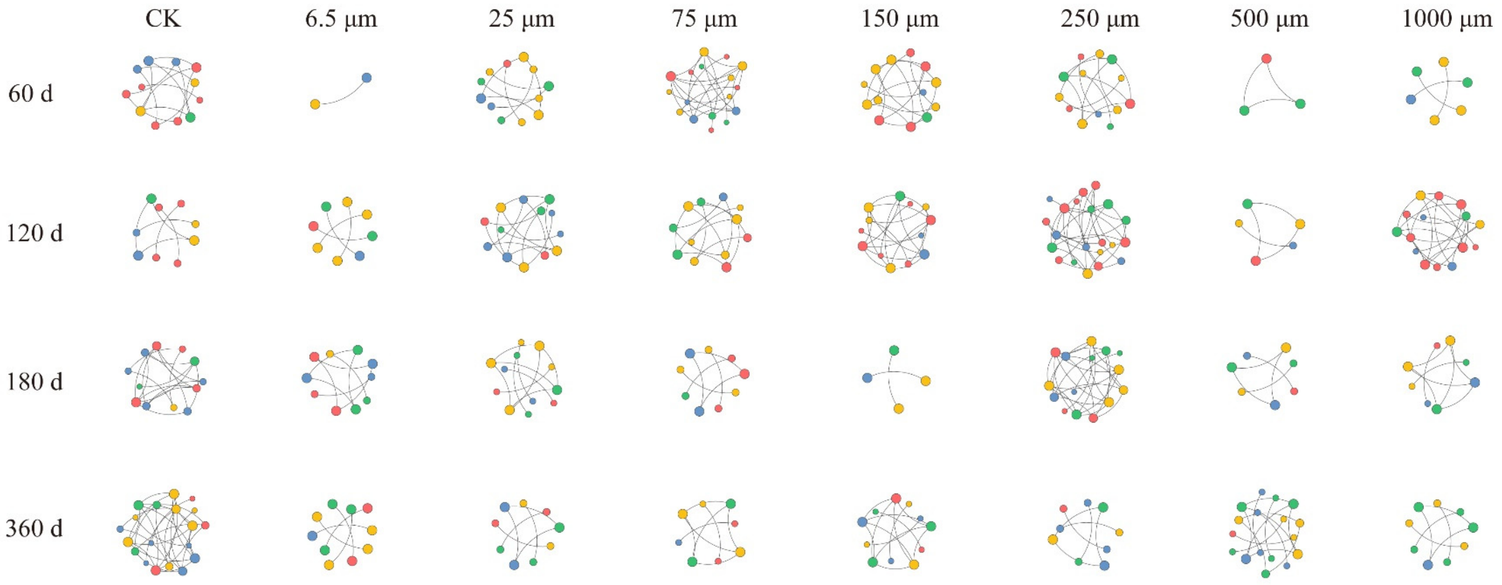


**Figure S2.** Co-occurrence network analysis of soil nematode communities across different treatments and sampling times. Red, yellow, blue, and green represent herbivores, bacterivores, fungivores, and omnivore-predators, respectively.

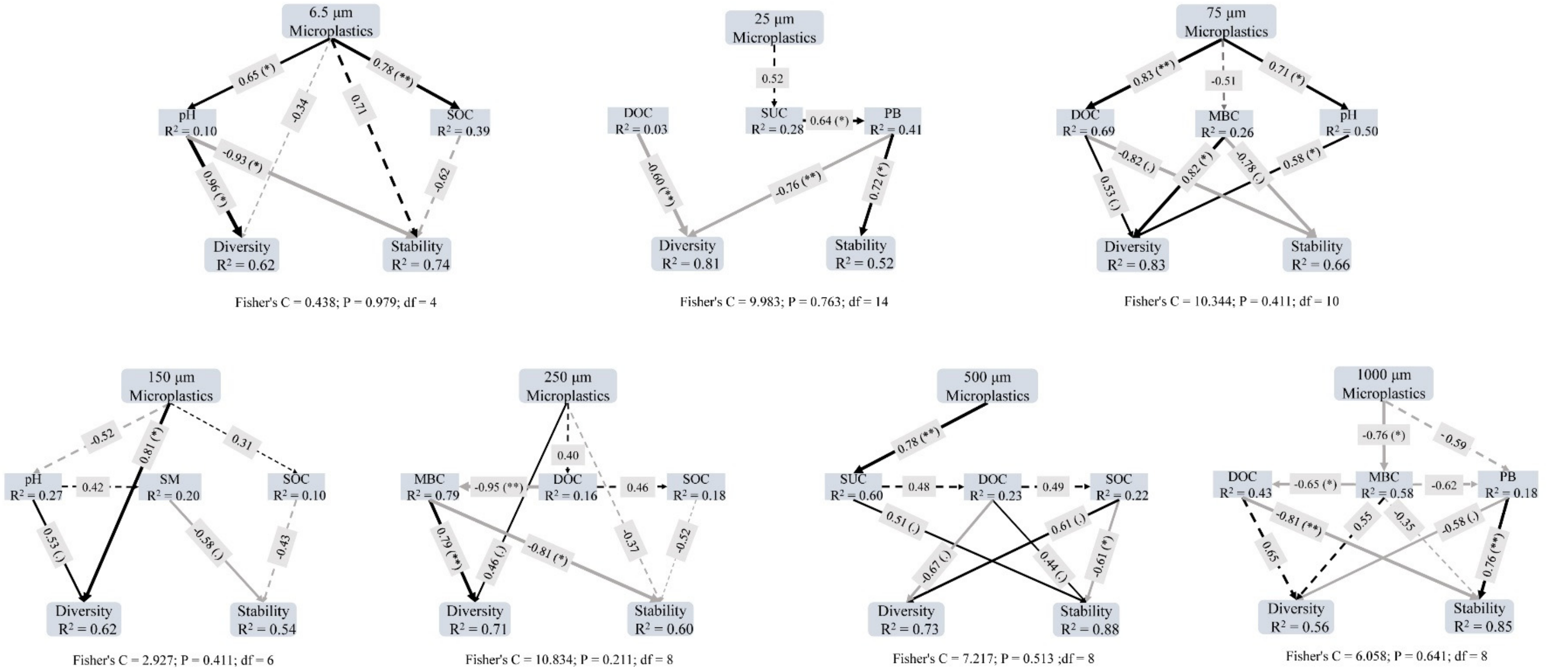


**Figure S3.** Structural equation modeling revealed divergent pathways through which microplastics of different particle sizes regulate nematode community diversity and stability. (.): $p < 0.1$, *: $p < 0.05$, and **: $p < 0.01$.

**Table S1.** Taxonomic composition of soil nematode communities at the genus level under different treatments.

| Genus | CK | 6.5 μm | 25 μm | 75 μm | 150 μm | 250 μm | 500 μm | 1000 μm |
|---|---|---|---|---|---|---|---|---|
| *Coslenchus* | 0.40±0.99 | 0.25±0.55 | / | 0.15±0.49 | 0.25±0.64 | 0.35±1.35 | / | 0.40±0.88 |
| *Boleodorus* | 0.05±0.22 | / | / | 0.05±0.22 | 0.05±0.22 | / | / | / |
| *Basiria* | / | / | / | 0.05±0.22 | / | 0.10±0.45 | 0.05±0.22 | 0.10±0.45 |
| *Aglenchus* | 0.15±0.49 | / | / | / | / | 0.05±0.22 | / | / |
| *Malenchus* | 0.05±0.22 | / | / | / | 0.05±0.22 | 0.05±0.22 | / | / |
| *Filenchus* | 1.00±1.52 | 1.45±1.85 | 1.50±2.82 | 3.25±3.46 | 1.75±2.02 | 1.20±1.79 | 0.95±2.33 | 1.60±2.46 |
| *Merlinius* | / | 0.15±0.49 | / | 0.25±1.12 | / | 0.05±0.22 | / | / |
| *Nagelus* | / | / | / | / | 0.15±0.49 | / | / | / |
| *Helicotylenchus* | 51.10±30.27 | 53.2±23.68 | 49.4±24.13 | 45.20±25.04 | 59.6±26.13 | 49.8±32.58 | 59.75±50.26 | 42.1±14.38 |
| *Pararotylenchus* | 0.15±0.67 | / | / | / | 0.10±0.45 | 0.05±0.22 | / | 0.05±0.22 |
| *Rotylenchus* | / | / | / | 0.05±0.22 | 0.15±0.49 | / | / | / |
| *Rotylenchulus* | 0.20±0.70 | 0.35±1.09 | 0.10±0.45 | 0.20±0.70 | 0.30±1.34 | 0.25±0.79 | / | 0.25±1.12 |
| *Criconemella* | / | / | / | / | / | 0.05±0.22 | / | 0.05±0.22 |
| *Criconemoides* | 0.10±0.31 | 0.05±0.22 | 0.10±0.45 | 0.05±0.22 | 0.15±0.49 | 0.05±0.22 | 0.05±0.22 | 0.10±0.31 |
| *Macroposthonia* | / | 0.05±0.22 | 0.35±1.18 | / | 0.15±0.49 | 0.10±0.45 | / | / |
| *Patatylenchus* | 50.45±24.53 | 51.5±37.83 | 47.95±20.17 | 40.95±28.05 | 33.65±20.81 | 43.6±28.65 | 59.15±43.12 | 44.85±20.18 |
| *Ditylenchus* | / | 0.20±0.89 | 0.10±0.45 | 0.10±0.45 | 0.45±1.39 | 0.05±0.22 | 0.15±0.67 | / |
| *Nothotylenchus* | 0.05±0.22 | 0.05±0.22 | 0.05±0.22 | 0.40±1.19 | 0.05±0.22 | 0.35±1.35 | 0.15±0.37 | 0.10±0.31 |
| *Pseudhalenchus* | / | 0.15±0.49 | 0.30±1.34 | / | / | / | / | 0.05±0.22 |
| *Aphelenchus* | 0.40±0.94 | 1.05±2.52 | 0.65±1.69 | 0.60±1.5 | 0.35±1.57 | 0.95±2.31 | 0.75±1.71 | 1.30±3.87 |
| *Paraphelenchus* | 0.70±1.45 | 0.95±1.50 | 1.05±1.99 | 0.95±1.67 | 0.65±0.88 | 0.45±0.76 | 0.70±0.86 | 0.30±0.73 |
| *Aprutides* | 0.15±0.49 | 0.15±0.49 | / | 0.15±0.49 | 0.05±0.22 | 0.10±0.31 | 0.10±0.31 | 0.10±0.31 |
| *Aphelenchoides* | 2.85±3.57 | 3.60±4.73 | 1.65±1.76 | 3.70±4.32 | 2.20±2.91 | 1.60±2.11 | 1.80±2.04 | 1.55±2.04 |

| | | | | | | | | |
|---|---|---|---|---|---|---|---|---|
| *Seinura* | / | / | / | / | 0.05±0.22 | 0.10±0.45 | / | / |
| *Rhabditis* | 0.50±1.10 | 0.95±1.93 | 0.45±0.89 | 0.65±0.99 | 0.30±0.8 | 0.25±0.72 | 0.35±0.67 | 0.50±1.32 |
| *Pellioditis* | 0.10±0.31 | / | / | / | / | / | / | / |
| *Rhabditis* | 0.40±1.10 | 0.20±0.52 | 0.25±0.72 | 0.15±0.49 | 0.50±1.79 | 0.20±0.52 | 0.10±0.45 | 0.35±0.93 |
| *Rhabditophanes* | 2.10±3.63 | 1.45±2.39 | 1.30±2.68 | 5.25±10.49 | 2.75±8.24 | 1.35±2.28 | 1.65±4.07 | 2.35±5.71 |
| *Bunonema* | / | / | / | 0.15±0.67 | 0.05±0.22 | / | / | / |
| *Cephalobus* | 0.25±0.91 | 0.10±0.31 | 0.10±0.31 | 0.60±2.46 | 0.30±1.13 | 0.30±0.92 | 0.25±0.79 | 0.40±1.19 |
| *Eucephalobus* | 2.95±3.71 | 3.90±6.09 | 3.35±5.77 | 4.60±7.51 | 4.05±7.23 | 2.45±2.39 | 2.50±4.56 | 3.65±7.76 |
| *Acrobeloides* | 0.20±0.41 | 0.85±2.01 | 0.95±1.64 | 0.15±0.37 | 0.25±0.55 | 0.30±0.73 | 0.20±0.52 | 0.45±1.10 |
| *Acrolobus* | / | / | / | / | / | / | 0.10±0.31 | / |
| *Panagrolaimus* | / | 0.10±0.31 | / | 0.05±0.22 | / | 0.05±0.22 | / | / |
| *Teratocephalus* | / | / | 0.05±0.22 | / | 0.05±0.22 | / | / | / |
| *Butlerius* | / | 0.05±0.22 | / | / | / | / | / | 0.10±0.45 |
| *Monhystera* | 0.35±1.57 | 0.85±2.11 | 0.20±0.70 | 0.35±1.35 | 0.15±0.49 | 1.65±4.99 | / | 0.25±0.79 |
| *Eumonhystera* | 0.10±0.31 | / | / | / | 0.05±0.22 | / | / | / |
| *Plecyus* | / | 0.10±0.31 | / | / | 0.05±0.22 | / | 0.05±0.22 | 0.05±0.22 |
| *Chronogaster* | / | / | 0.05±0.22 | 0.15±0.67 | 0.05±0.22 | 0.05±0.22 | 0.05±0.22 | 0.05±0.22 |
| *Achromadora* | / | 0.05±0.22 | / | / | / | / | / | 0.10±0.31 |
| *Prodesmodora* | 0.10±0.31 | 0.05±0.22 | / | 0.25±1.12 | 0.30±0.98 | 0.25±0.72 | / | 0.20±0.52 |
| *Odontolaimus* | / | / | 0.05±0.22 | / | / | / | / | / |
| *Prismatolaimus* | 0.05±0.22 | / | / | 0.05±0.22 | / | / | 0.05±0.22 | 0.20±0.52 |
| *Tripyla* | 0.15±0.67 | / | / | 0.05±0.22 | 0.45±1.47 | 0.15±0.67 | 0.05±0.22 | / |
| *Trischistoma* | / | / | 0.05±0.22 | / | / | 0.05±0.22 | / | / |
| *Alaimus* | 0.05±0.22 | 0.35±0.67 | 0.25±0.55 | 0.05±0.22 | 0.25±0.72 | 0.50±1.00 | 0.30±0.98 | 0.15±0.37 |
| *Mylonchulus* | 1.20±1.36 | 1.25±1.62 | 0.50±0.89 | 1.45±2.37 | 1.10±1.74 | 1.4±1.98 | 0.85±1.50 | 1.35±3.41 |
| *Chrysonemoides* | / | / | 0.15±0.49 | / | 0.10±0.31 | / | 0.15±0.67 | 0.15±0.49 |

| | | | | | | | | |
|---|---|---|---|---|---|---|---|---|
| *Prodorylaimium* | 3.10±3.60 | 4.00±3.61 | 4.65±5.62 | 8.45±8.89 | 7.70±9.08 | 6.45±4.94 | 3.85±3.54 | 6.05±5.46 |
| *Mesodorylaimus* | 3.40±4.68 | 3.25±4.84 | 4.45±6.32 | 3.20±5.21 | 4.20±5.11 | 2.35±4.42 | 3.20±4.54 | 2.80±3.58 |
| *Enchodelus* | 0.80±3.58 | 0.05±0.22 | 0.05±0.22 | 0.20±0.70 | 0.05±0.22 | 0.30±1.34 | / | 0.65±2.68 |
| *Thonus* | 0.35±1.14 | 0.10±0.31 | 0.35±1.18 | 0.15±0.49 | / | 0.05±0.22 | 0.50±1.28 | / |
| *Allodorylaimus* | 0.85±1.50 | 0.90±2.00 | 0.95±1.93 | 0.65±1.42 | 1.25±2.4 | 1.00±1.72 | 0.85±2.18 | 1.10±2.79 |
| *Epidorylaimus* | 1.00±1.69 | 2.05±4.98 | 0.70±1.69 | 1.50±3.14 | 1.75±4.08 | 1.90±3.70 | 0.55±1.23 | 0.75±1.94 |
| *Aporcelaimellus* | 3.95±4.52 | 7.30±9.19 | 4.25±4.80 | 5.85±6.50 | 7.75±10.23 | 5.35±4.72 | 4.00±4.30 | 5.55±12.04 |
| *Dorylaimoides* | 0.20±0.41 | 0.25±0.64 | 0.40±1.14 | 0.10±0.31 | 0.35±0.88 | 0.15±0.49 | 0.25±0.72 | 0.20±0.52 |
| *Tylencholaimus* | 0.60±1.47 | 0.25±0.64 | 0.10±0.31 | 0.25±0.44 | 0.40±1.1 | 0.30±0.66 | 0.30±0.80 | 0.85±3.34 |

**Table S2.** Means ± SD of soil nematode community metrics under different microplastic particle sizes and sampling times.

| Sampling time | Measured metrics | CK | 6.5 μm | 25 μm | 75 μm | 150 μm | 250 μm | 500 μm | 1000 μm |
|---|---|---|---|---|---|---|---|---|---|
| 60 | Abundance | 446.63±185.42 | 522.26±381.08 | 737.34±234.39 | 468.74±121.03 | 626.42±172.5 | 528.42±206.44 | 488.76±214.96 | 583.59±298.31 |
| 60 | Richness | 11.6±4.04 | 11±2.45 | 10±1.58 | 9.6±2.07 | 13±2.92 | 11.8±2.49 | 8.8±0.84 | 10.8±2.77 |
| 60 | Shannon-Wiener index | 1.55±0.46 | 1.69±0.37 | 1.55±0.27 | 1.57±0.52 | 1.67±0.28 | 1.7±0.23 | 1.36±0.19 | 1.46±0.24 |
| 60 | Evenness index | 0.43±0.06 | 0.51±0.07 | 0.49±0.12 | 0.53±0.15 | 0.43±0.11 | 0.48±0.07 | 0.45±0.1 | 0.43±0.11 |
| 60 | Herbivores (%) | 77.47±13.9 | 69.78±12.9 | 73.45±10.74 | 73.19±18.66 | 69.14±15.67 | 69.01±12.83 | 79.46±6.79 | 76±19.45 |
| 60 | Omnivores-predators (%) | 8.89±7.5 | 7.25±3.54 | 6.9±3.7 | 7.92±7.38 | 15.61±10.01 | 13.05±6.77 | 7.93±4.75 | 7.35±5.15 |
| 60 | Bacterivores (%) | 9.08±6.61 | 15.29±9.1 | 12.39±5.89 | 13.09±11.16 | 11.07±12.89 | 13.25±6.25 | 7.51±5.12 | 13.76±12.92 |
| 60 | Fungivores (%) | 4.56±1.6 | 7.68±4.98 | 7.25±3.6 | 5.81±3.94 | 4.18±1.34 | 4.69±3.11 | 5.1±1.28 | 2.9±1.98 |
| 120 | Abundance | 1456.76±833.85 | 1254.42±1059.64 | 1110.87±573.84 | 1062.2±879.51 | 1000.12±740.12 | 1484.99±892.11 | 1454.18±824.93 | 803.53±570.53 |
| 120 | Richness | 8.2±2.39 | 9.6±1.14 | 8.6±2.88 | 10±1.58 | 10.2±0.84 | 9±3.16 | 6.8±1.64 | 8.4±2.51 |
| 120 | Shannon-Wiener index | 1.15±0.35 | 1.28±0.26 | 1.09±0.43 | 1.56±0.41 | 1.42±0.1 | 1.22±0.39 | 0.91±0.17 | 1.22±0.32 |
| 120 | Evenness index | 0.41±0.08 | 0.38±0.07 | 0.46±0.11 | 0.5±0.14 | 0.41±0.05 | 0.41±0.11 | 0.38±0.09 | 0.42±0.08 |
| 120 | Herbivores (%) | 85.55±5.78 | 85.12±8.12 | 82.96±14.29 | 74.31±15.18 | 79.08±8.18 | 86.05±12.42 | 93.26±5.09 | 86.46±9.36 |

| | | | | | | | | | |
|---|---|---|---|---|---|---|---|---|---|
| 120 | Omnivores-predators (%) | 4.72±4.62 | 5.2±1.95 | 8.71±8.2 | 11.99±10.03 | 12.28±10.09 | 8.15±10.74 | 3.14±2.32 | 8.09±6.5 |
| 120 | Bacterivores (%) | 5.22±5.03 | 6.48±5.96 | 5.43±5.37 | 7.4±7.61 | 5.14±2.73 | 2.85±2.95 | 1.79±2.3 | 2.94±1.67 |
| 120 | Fungivores (%) | 4.51±3.52 | 3.2±3.99 | 2.91±2.47 | 6.29±3.72 | 3.49±1.62 | 2.94±3.55 | 1.81±1.32 | 2.51±2.97 |
| 180 | Abundance | 1088.11±797.43 | 1143.33±640.66 | 897.84±438.55 | 918.47±452.12 | 830.88±434.39 | 1073.02±807.78 | 1101.74±506.92 | 676.05±350.38 |
| 180 | Richness | 7.4±1.67 | 9.2±2.59 | 9±3.08 | 9.4±2.07 | 7.4±2.07 | 8.8±2.68 | 9.6±3.78 | 8.8±3.11 |
| 180 | Shannon-Wiener index | 1.19±0.35 | 1.17±0.34 | 1.25±0.29 | 1.39±0.44 | 1.22±0.24 | 1.32±0.44 | 1.08±0.14 | 1.38±0.12 |
| 180 | Evenness index | 0.46±0.08 | 0.37±0.05 | 0.41±0.07 | 0.45±0.12 | 0.5±0.16 | 0.45±0.09 | 0.34±0.12 | 0.49±0.11 |
| 180 | Herbivores (%) | 87.77±11.68 | 87.81±8.22 | 88.17±7.14 | 79.58±14.56 | 83.81±10.69 | 83.52±9.28 | 90.13±4.35 | 82.82±6.01 |
| 180 | Omnivores-predators (%) | 8.61±7.86 | 9.21±5.31 | 8.7±4.5 | 14.4±9.88 | 14.13±9.29 | 13.56±5.89 | 6.51±4.15 | 11.67±3.67 |
| 180 | Bacterivores (%) | 0.53±1.18 | 0.64±0.87 | 1.48±1.94 | 1.27±0.91 | 0.31±0.44 | 0.7±0.98 | 0.89±0.76 | 1.89±1.75 |
| 180 | Fungivores (%) | 3.1±3.18 | 2.35±3.68 | 1.65±1.93 | 4.76±4.66 | 1.74±1.75 | 2.23±3.34 | 2.47±1.91 | 3.62±5.62 |
| 360 | Abundance | 1883.37±475.69 | 1737.26±542.65 | 1569.1±493.74 | 1617.92±641.86 | 1577.29±396.06 | 1718.1±727.68 | 1580.19±533.33 | 1241.4±861.62 |
| 360 | Richness | 13.4±3.36 | 14.4±2.61 | 11.4±4.22 | 14.2±2.77 | 13.6±2.19 | 13.4±3.36 | 13±4.36 | 12.6±3.58 |
| 360 | Shannon-Wiener index | 1.73±0.34 | 1.94±0.35 | 1.61±0.44 | 2.02±0.28 | 1.91±0.18 | 1.95±0.47 | 1.81±0.33 | 1.9±0.45 |
| 360 | Evenness index | 0.44±0.04 | 0.5±0.13 | 0.47±0.03 | 0.55±0.12 | 0.51±0.09 | 0.58±0.13 | 0.49±0.06 | 0.56±0.13 |
| 360 | Herbivores (%) | 68.9±11.4 | 57.72±12.6 | 71.41±15.95 | 49.91±15.51 | 52.33±18.25 | 57.57±21.66 | 63.64±12.93 | 54.92±18.24 |
| 360 | Omnivores-predators (%) | 20.49±9.69 | 30.52±9.6 | 21.57±11.67 | 29.53±6.24 | 32.05±10.26 | 30.6±19.27 | 25.58±7.77 | 31.44±16.19 |
| 360 | Bacterivores (%) | 7.35±3.22 | 5.9±2.25 | 4.28±2.31 | 16.36±9.49 | 11.48±10.48 | 7.72±2.07 | 7.16±6.06 | 8.99±7.25 |
| 360 | Fungivores (%) | 3.27±2.08 | 5.86±4.75 | 2.75±2.7 | 4.2±2.77 | 4.14±3.02 | 4.12±4.3 | 3.62±1.82 | 4.65±5.75 |

**Table S3.** Mean ± SD of environmental factors under different microplastic particle size treatments.

| Factors | CK | 6.5 μm | 25 μm | 75 μm | 150 μm | 250 μm | 500 μm | 1000 μm |
|---|---|---|---|---|---|---|---|---|
| MBC (mg/kg) | 366.66±21.44 | 362.09±12.8 | 358.89±20.53 | 308.7±24.71 | 346.91±22.61 | 347.76±31.47 | 341.85±22.71 | 283.67±8.4 |
| DOC (mg/kg) | 262.07±28.01 | 262.45±25.38 | 261.34±30.36 | 332.25±23.98 | 307.87±41.92 | 284.58±30.25 | 286.11±38.11 | 295.04±36.34 |
| SOC (g/kg) | 12.8±0.53 | 14.13±0.67 | 14.39±1.04 | 12.94±0.51 | 13.16±0.72 | 12.83±0.75 | 12.9±0.33 | 12.55±0.49 |

| | | | | | | | | |
|---|---|---|---|---|---|---|---|---|
| MBN (mg/kg) | 28.96±4.26 | 30.4±5.72 | 29.5±4.28 | 31.72±5.87 | 30.17±6.02 | 27.87±7.98 | 25.35±3.65 | 28.87±5.27 |
| DON (mg/kg) | 41.29±5.37 | 40.14±4.16 | 41.23±4.9 | 44.34±6.26 | 41.55±4.64 | 42.51±5.36 | 45.83±5.66 | 40.48±4.81 |
| TN (mg/kg) | 640.53±34.03 | 618.28±22.96 | 625.05±43.79 | 641.61±35.7 | 621.29±24.5 | 609.96±19.78 | 637.63±33.99 | 612.3±18.53 |
| SUC (mg/kg) | 78.18±11.15 | 86.14±23.31 | 100.46±26.39 | 89.38±24 | 78.24±14.61 | 91.7±25.99 | 100.3±8.87 | 81.9±14.41 |
| URE (mg/kg) | 427.74±25.26 | 466.46±70.32 | 472.42±57.64 | 460.96±67.47 | 432.68±84.84 | 438.66±72.38 | 457.92±78.16 | 418.8±26.69 |
| PLA (mg/kg) | 379.38±39.21 | 289.76±99.19 | 278.42±69.46 | 271.58±79.28 | 282.76±84.8 | 294.86±67.65 | 320.12±98.25 | 263.62±55.71 |
| SM (%) | 21.28±1.25 | 21.15±1.39 | 21.67±1.19 | 21.48±1.43 | 21.21±1.38 | 21.33±1.39 | 20.54±1.32 | 21.31±1.32 |
| pH | 5.28±0.11 | 5.45±0.11 | 5.21±0.25 | 5.55±0.18 | 5.18±0.06 | 5.4±0.31 | 5.23±0.16 | 5.46±0.18 |
| PB (g) | 2598.4±508.78 | 2322±872.74 | 2566±547.92 | 2188.8±787.99 | 2603.33±599.77 | 2506±350.19 | 2956±418.19 | 2480.67±546.51 |

**Table S4.** Explanation of environmental factors for the variation of soil nematode community composition

| Factors | Explanatory (%) | *P* |
|---|---|---|
| MBC | 20.64 | 0.008 |
| PB | 14.46 | 0.045 |
| ALP | 4.63 | 0.098 |
| DON | 3.61 | 0.143 |
| SM | 3.12 | 0.158 |
| pH | 3.09 | 0.159 |
| DOC | 2.82 | 0.144 |
| TN | 1.63 | 0.198 |
| SOC | 1.42 | 0.237 |
| SUC | 1.01 | 0.376 |
| URE | 0.69 | 0.469 |
| MBN | 0.52 | 0.577 |
